# Effect of Water Level and Tannin Inclusion on In Vitro Degradability and Digestibility of Soybean Meal

**DOI:** 10.3390/ani16050718

**Published:** 2026-02-25

**Authors:** Nejc Valcl, Andrej Lavrenčič

**Affiliations:** Biotechnical Faculty, University of Ljubljana, 1000 Ljubljana, Slovenia; andrej.lavrencic@bf.uni-lj.si

**Keywords:** in vitro, bypass protein, soybean meal, tannins, ruminant

## Abstract

Optimizing dietary protein utilization is essential for improving ruminant nutrition, and tannins can reduce the ruminal degradability of soybean meal (SBM) proteins through tannin–protein complex formation, a process enhanced by water. This study evaluated three water levels (1:0.625, 1:1.25, 1:2.5 *w*/*v*) combined with chestnut or quebracho tannins at 50 and 100 g/kg on in vitro dry matter (DM) and crude protein (CP) degradability and digestibility of SBM. Increasing water levels significantly reduced crude protein degradability (*p* < 0.05), with the greatest decline observed for chestnut tannins at 100 g/kg, decreasing from 640 g/kg at the lowest water level to 423 g/kg at the highest. Post-ruminal crude protein digestibility increased slightly with water for this treatment (from 977 to 984 g/kg). Bypass protein content ranged from 138 g/kg (quebracho 100 g/kg, low water) to 563 g/kg (chestnut 100 g/kg, high water), with increases of 20.7% and 22.6% for chestnut tannins at 50 and 100 g/kg, respectively. Bypass protein digestibility improved by up to 4.4%. Dry-matter degradability decreased by 6.8% to 23.5% depending on treatment. These findings demonstrate that water greatly enhances tannin efficacy and highlight its potential for improving protein utilization.

## Simple Summary

Efficient use of dietary protein is necessary for healthy and sustainable ruminant production. Soybean meal is a common protein source for ruminants, but it is broken down too quickly in the rumen, leading to nitrogen losses for the animal. One way to offset this effect is with the addition of tannins, which bind protein making them less degradable in the rumen. The binding process is affected by water. The aim of this study was to assess the effects of low, medium and high water levels combined with two tannins, added at two concentrations on the in vitro degradability and digestibility of dry matter and crude protein in soybean meal. The results indicate that higher water levels effectively reduce rumen protein degradation and increase protein available for post-ruminal digestion, with no effect on its digestibility, regardless of tannin type or concentration. Rumen degradation of dry matter was reduced as water and tannins increased, which could negatively affect short-chain fatty acid production, rumen microbiota and consequently rumen fermentation. Overall, optimizing water and tannin levels during feed processing may improve the supply of usable protein to ruminants, but fermentation impacts need further research.

## 1. Introduction

Improving feed conversion efficiency and growth rate while reducing air and water pollution from nitrogen losses through emissions of urea, ammonia and nitrous oxide is a goal for sustainable livestock production [1]. Moreover, high ruminal degradability of protein-rich feeds, including soybean meal (SBM), reduces the biological value of the protein and leads to increased nitrogen losses [2]. As a staple protein feed in both dairy and beef cattle, SBM contains a favourable amino acid profile and high crude protein content [3]. However, SBM also has a high ruminal degradability which negatively impacts the environment and post-ruminal amino acid availability due to inefficient ruminal utilization [4]. Improving ruminal undegradable protein (bypass protein) can enhance metabolizable protein supply to the small intestine, thereby supporting animal performance while reducing nitrogen excretion [5]. A variety of strategies exist for reducing the rumen protein degradation of protein feeds, one of which is including bioactive phytochemicals such as tannins into the diet of ruminants [6].

Tannins are a group of complex polyphenols, classified according to their chemical structure as hydrolysable (HT) and condensed tannins (CT) [7]. They are used as feed additives in ruminant nutrition able to inhibit cellulolytic and proteolytic activities in the rumen, affecting methane emissions and urine nitrogen excretion [8]. Tannins form tannin–protein complexes with dietary protein in protein feeds, reducing the availability of dietary protein for rumen microbiota [9]. When used in ruminant nutrition, tannins have also shown negative effects such as reduced voluntary feed intake as well as reduced nutrient digestibility, showing possible harm to ruminant production [7]. One of the primary reasons for tannin negative effects in ruminant nutrition is the need for relatively high tannin inclusion rates to achieve sufficient protein protection [10]. Tannin–protein complex formation is strongly influenced by tannin type, as HT and CT differ in their effect as ruminant feed additives. Hydrolysable tannins bind protein more strongly and are degraded in the rumen into compounds that are absorbed beyond the rumen [11]. On the other hand, CT can directly affect the degradation of carbohydrates, bind to microbial nutrients and enzymes and weaken attachment of microbes to feed particles [12].

In ruminant nutrition, chestnut wood extract (*Castanea sativa* L.) is a common source of HT, while quebracho wood extract (*Schinopsis* spp.) is widely used as a source of CT. Differences between these two tannin sources were documented in previous studies in SBM. Lavrenčič and Levart [13] demonstrated that chestnut wood extract tannins (CWE) reduced crude protein degradability in SBM at lower inclusion levels than quebracho wood extract (QUE). Similarly, Li et al. [14] reported that fermented SBM treated with CWE showed a stronger increase in rumen undegradable protein than QUE. Tannin concentration and substrate type are also important factors in tannin–protein complex formation. Secondary treatment conditions, such as the presence of heat and water, have recently also gained importance in optimizing SBM protein protection. Water acts as a chemical facilitator for binding tannin molecules to proteins, as the primary mechanism for complex formation are hydrophobic interactions and hydrogen bonding [15,16]. This was shown by recent SBM studies where the presence of water in tannin–protein feed mixtures catalyzed the binding between tannins and proteins, having a stronger effect on the reduction in crude protein ruminal degradability [13,17,18].

While the presence of water is chemically required for tannin–protein interactions, the quantity of water used during feed treatment represents a technological process variable that may influence the efficiency, extent, and uniformity of complex formation. Currently, no clear specifications on the optimal water quantity for tannin–protein complexation are available in the existing literature. The amounts of water added reported in the literature ranged from spraying tannins mixed with water on the substrate to completely submerging the tannin–protein feed mixture, making comparisons between studies difficult. Without clear guidelines, standardization of tannin–protein treatments are challenging, leading to inconsistent results across studies and limiting their application in practice. Reducing water content in the process could also offer economic, environmental and practical benefits, such as reduced energy and water costs, lowering the environmental impact of the treatment process, speeding up protein precipitation and subsequent drying, consequently leading to faster processing times [19]. Understanding how water quantity influences tannin–protein complex formation is therefore essential for optimizing SBM processing and potentially reducing the need for high tannin inclusion rates.

While previous studies have demonstrated that water facilitates tannin–protein binding, no systematic evaluation of water quantity in combination with different tannin types and concentrations has been conducted. This study is also a continuation of a previous experiment, where Valcl and Lavrenčič [17] have shown that efforts in optimizing the drying process of tannin–protein SBM mixtures can improve ruminal undegradable protein without impairing digestibility. Therefore, the specific aim of this study was to evaluate how three quantities of added water (low, medium, high) interact with two tannin sources (chestnut and quebracho), each applied at two inclusion levels (50 and 100 g/kg), in order to identify combinations that most effectively increase ruminal protein protection in SBM without impairing post-ruminal digestibility. By clarifying the role of water in tannin–protein complexation, this study may contribute to development of feed processing strategies that enhance the efficiency of dietary protein utilization and improve the nutritional value of SBM for ruminants.

## 2. Materials and Methods

### 2.1. Ethical Approval

All animal procedures were approved by the Slovenian Ministry of Agriculture (Permit No. U34401-5/2024/10 dated 3 July 2024, issued by the Inspection for Food Safety, Veterinary Sector and Plant Protection, Administration of the Republic of Slovenia for Food Safety, Veterinary Sector and Plant Protection, Ministry of Agriculture, Forestry and Food, Ljubljana, Slovenia).

### 2.2. Sample Preparation

Chestnut water extract (CWE) tannins (Farmatan, Tanin Sevnica, Sevnica, Slovenia) were obtained from Tanin Sevnica and quebracho (QUE) tannins (Tannino Red Plus, Tecnofood Italia, Santa Maria della Versa, Italy) were added to SBM according to the modified sample preparation of Valcl and Lavrenčič [17]. In brief: 100 g of SBM mixed with 50 or 100 g/kg of CWE and QUE were added to plastic containers. The tannin contents (76% for CWE and 70% for QUE, DM basis) correspond to manufacturer declared values reported in the official product specifications. Distilled water was then added at three water levels: low water level (LW) of 62.5 mL (1:0.625 *w*/*v*), medium water level (MW) of 125 mL (1:1.25) and high water level (HW) of 250 mL (1:2.5). The material was then stirred manually to obtain a homogeneous mixture, covered with aluminum foil to prevent excessive evaporation, and stored at 20 °C for 24 h. The samples were dried at 80 °C in an air-forced oven for 48 h, ground to 1 mm using a knife mill (Grindomix GM 300, Retsch GmbH, Haan, Germany) and stored until further analysis. SBM without added tannins but subjected to the same treatment was included as a control sample for each water level. Each sample was created in duplicate batches, obtaining a total of 40 samples. Samples were analyzed for dry matter (DM), crude protein (CP), ether extract (EE) and ash. Neutral detergent fibre (NDF) and acid detergent fibre (ADF) were analyzed with the procedure of Van Soest et al. [20]. The chemical compositions of all samples are presented in Table 1.

### 2.3. Experimental Design and In Vitro Measurement of Dry Matter and Crude Protein Degradability and Digestibility

Rumen fluid was collected from three adult, castrated and cannulated Jezersko Solčavska × Romanovska rams (*Ovis aries*) with an average weight of 70 kg. This number of donor animals was used to obtain a representative microbial inoculum, as is standard in in vitro rumen studies, while minimizing unnecessary animal use [21,22,23]. The rams were obtained from the Educational Research Centre for Animal Husbandry Logatec (Biotechnical Faculty, University of Ljubljana, Slovenia). They were offered approx. 1.5 kg daily matter intake of medium quality hay ad libitum (*Lolium perenne*), obtained from a permanent grassland with the following composition: ash = 38 g/kg DM, CP = 172 g/kg DM, NDF = 579 g/kg DM. This ration was supplemented daily by 250 g of pelleted commercial compound feed containing 160 g CP per kg DM, 50 g of SBM, and 25 g of mineral–vitamin mixture to ensure that the donor animals maintained an active and functionally diverse rumen microbiota capable of degrading starch-, protein-, and fibre-rich substrates. No feed additives were included in the basal diet. The basal diet was formulated based on the German standards for metabolizable energy and utilizable protein requirements [24].

Measurements of in vitro crude protein degradability and digestibility were performed using the ANKOM Technology DAISY II incubator (ANKOM Technology, Macedon, NY, USA). Rumen fluid was collected before morning feeding and was transported to the laboratory in a pre-warmed thermos bottle (39 °C). The following procedure was performed with constant CO_2_ flushing. The rumen fluid was strained through four layers of cheesecloth and transferred to a jar containing a prepared buffer solution [25]. The buffer solution was prepared before collection of rumen fluid. The total volume of the inoculum was 2 litres and the ratio of rumen fluid volume of buffer solution volume was 1:4. Approximately 4 g of each substrate (tannin × concentration × water) was weighed into four R510 concentrate bags (size: 5 × 10 cm; porosity: 50 ± 10 µm; ANKOM Technology, Macedon, NY, USA). Each substrate was prepared in duplicate batches which totalled to eight bags per substrate over the course of the experiment, and each bag was considered as a technical replicate. The substrate quantity per bag area was 40 mg/cm^2^ [13]. Substrates from each batch were incubated in two jars, meaning each treatment had two independent jar-level observations per run. The incubation jar served as the experimental unit. The bags were then incubated across two jars at 39 °C for 24 h with constant rotation. Each substrate was present in both jars and jar sides which totalled to 2 substrate bags per jar. Each jar contained a maximum of 4 substrates (16 bags/jar). Additionally, each incubation run contained two hay standard bags (*Lolium multiflorum*), and two blanks, totalling to 36 bags per run (number of runs = 5). The substrates were allocated equally across runs according to their batch (batch 1 = runs 1–3, batch 2 = runs 3–5). The hay standard bags were used to control and correct in vitro dry matter degradation between runs if the difference in hay standard between runs was higher than 5%.

After incubation, bags were rinsed under cold tap water until the wash water was clear. Bags were then oven-dried at 50 °C for 24 h, weighed, and in vitro dry matter degradability (ivDMDeg) was calculated. For digestibility determination, the three-step enzymatic procedure described by Gargallo et al. [26] was applied. Following rumen incubation, bags were incubated sequentially in pepsin–HCl (pH 1.9, 1 g pepsin/L) for 1 h and pancreatin–phosphate buffer (pH 7.75, 3 g pancreatin/L) for 24 h, both at 39 °C, respectively. Bags were then rinsed and dried as described above and in vitro dry matter digestibility (ivDMDig) was calculated.

A balanced factorial design with randomization in a 3 × 2 × 3 factorial arrangement was used. Substrate residues after incubation were pooled together according to in vitro determination (degradability or digestibility), type of tannin (NO, CWE, QUE), concentration of added tannin (50 or 100 g/kg), amount of water added (LW, MW, HW) and batch (1,2). The CP content was determined according to Naumann and Bassler [27] in the obtained substrates. In vitro crude protein degradability (ivCPDeg) and digestibility (ivCPDig) of substrates were calculated by subtracting the residual CP content after incubation from the CP content of the substrate. Bypass protein (CP_BP) was determined by subtracting ivCPDeg from total CP, and digestible bypass protein (dCP_BP) was calculated, following the approach of Cortés et al. [28], as: dCP_BP = CP_BP × ivCPDig.

### 2.4. Statistical Analysis

The data was analyzed in R (v4.5.1; R Core Team, 2025). A three-way analysis of variance (ANOVA) was performed to assess the effectiveness of tannin type (T), concentration (C), and water level (W), including all two-way and the three-way interaction on the response variables (ivDMDeg, ivDMDig, ivCPDeg, ivCPDig, CP_BP, dCP_BP). To determine which specific combination of tannin type, concentration and water level was responsible for the observed variation in the response variables, post hoc pairwise comparisons were conducted using the emmeans package (v2.0.1), employing Tukey’s method to adjust for multiple comparisons. Statistical significance was determined at *p* < 0.05 and the results are presented as least square means.

## 3. Results

The full numerical results of our examined variables can be found in Tables S1 and S2. The results of our three-way ANOVA are presented in Table 2. A significant T × C × W interaction was observed for ivCPDig (*p* = 0.006) and dCP_BP (*p* = 0.007), showing the influence of the combined effects of tannin type, concentration and water level on these parameters. The interactions between tannin type and concentration (T × C) and tannin type and water level (T × W) were both highly significant (*p* < 0.001) for all the analyzed variables except for dCP_BP (*p* = 0.157), while the interaction between tannin concentrations and water level (C × W) was insignificant in all variables.

**Figure 1:**
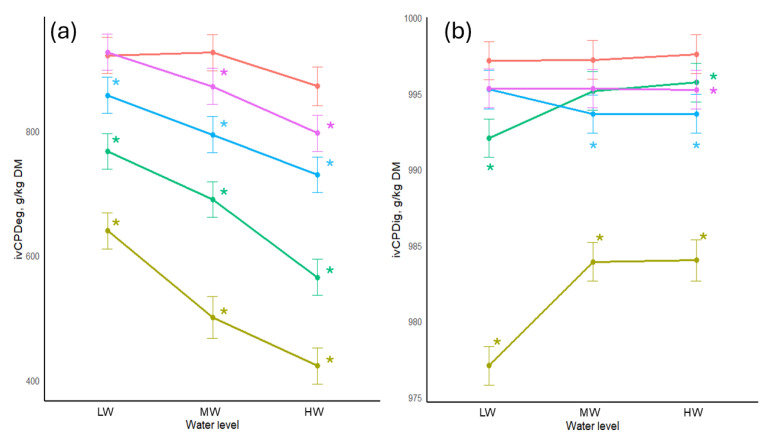
(**a**) In vitro crude protein degradability of soybean meal treated with tannins and different water levels. (**b**) In vitro crude protein digestibility of soybean meal treated with tannins and different water levels. Colours of the lines represent the amount and type of tannins added: red = no tannin added (control); purple = 50 g QUE/kg; blue = 100 g QUE/kg; green = 50 g CWE/kg; gold = 100 g CWE/kg. LW = water added at 1:0.6125 *w*/*v*; MW = water added at 1:1.25 *w*/*v*; HW = water added at 1:2.5 *w*/*v*; ivCPDeg—in vitro crude protein degradability; ivCPDig—in vitro crude protein digestibility. *—significant difference compared to the control of its respective water level.

In all figures presented below, the axes were scaled independently to enhance the visibility of treatment differences for each analyzed parameter. The ivCPDeg and ivCPDig of SBM treated with tannins and different water quantities are presented in Figure 1. The ivCPDeg was significantly decreased (*p* < 0.05) with increasing water levels, the highest reductions being at the HW. The largest decreases were observed when CWE at 100 g/kg were added. Values decreased from 640 g/kg DM at the low water level to 423 g/kg DM at the high level. Increasing water also significantly increased (*p* < 0.05) the ivCPDig for this treatment, rising from 977 g/kg DM at the low water level to 984 g/kg DM at both medium and high levels.

**Figure 2:**
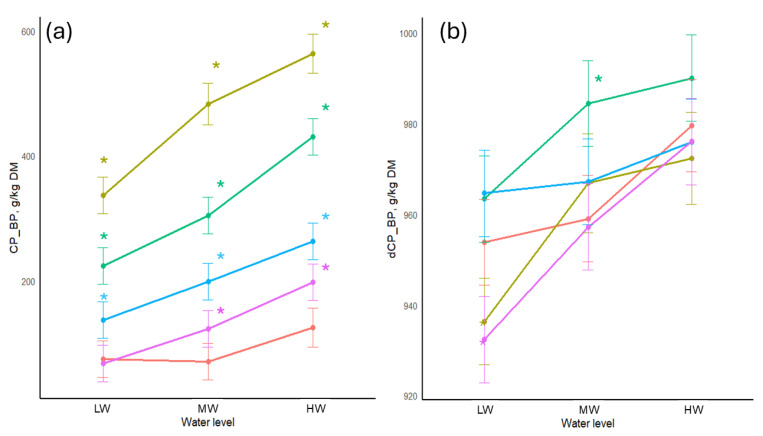
(**a**) Bypass crude protein content of soybean meal treated with tannins and different water levels. (**b**) Bypass crude protein digestibility of soybean meal treated with tannins and different water levels. Colours of the lines represent the amount and type of tannins added: red = no tannin added (control); purple = 50 g QUE/kg; blue = 100 g QUE/kg; green = 50 g CWE/kg; gold = 100 g CWE/kg. LW = water added at 1:0.6125 *w*/*v*; MW = water added at 1:1.25 *w*/*v*; HW = water added at 1:2.5 *w*/*v*; CP_BP—bypass crude protein content; dCP_BP—bypass crude protein digestibility. *—significant difference compared to the control of its respective water level.

The effects of combining tannins with different water levels on CP_BP and dCP_BP can be found in Figure 2. There were significant increases in CP_BP (*p* < 0.05) with the addition of tannins in both concentrations regardless of water level ranging from 138 g/kg DM when 100 g/kg QUE were added at the low water level to 563 g/kg DM when 100 g/kg CWE added at the high water level. Each instance of increased water level improved CP_BP, with the largest low-to-high water increases of 20.7% for CWE at 50 g/kg and 22.6% at 100 g/kg. Water level also significantly affected the dCP_BP for all treatments except 100 g/kg QUE. The greatest improvement was +4.4% for 50 g/kg QUE between the low and high water levels.

**Figure 3:**
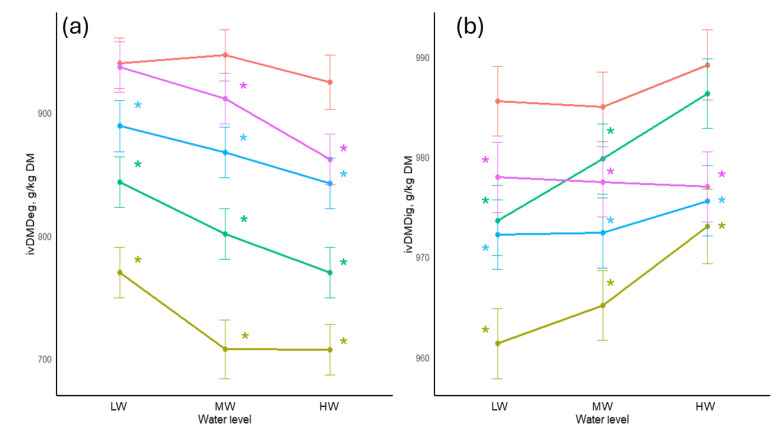
(**a**) In vitro dry matter degradability of soybean meal treated with tannins and different water levels. (**b**) In vitro dry matter digestibility of soybean meal treated with tannins and different water levels. Colours of the lines represent the amount and type of tannins added: red = no tannin added (control); purple = 50 g QUE/kg; blue = 100 g QUE/kg; green = 50 g CWE/kg; gold = 100 g CWE/kg. LW = water added at 1:0.6125 *w*/*v*; MW = water added at 1:1.25 *w*/*v*; HW = water added at 1:2.5 *w*/*v*; ivDMDeg—in vitro dry matter degradability; ivDMDig—in vitro dry matter digestibility. *—significant difference compared to the control of its respective water level.

The results for SBM treated with tannins and different water levels and their effects on ivDMDeg and ivDMDig are presented in Figure 3. There was a significant reduction (*p* < 0.05) in ivDMDeg. The largest reductions occurred at the highest water level, ranging from 6.8% for 50 g/kg QUE to 23.5% for 100 g/kg CWE. There was a gradual increase of 0.5% in ivDMDig when 50 g/kg CWE was added with each increased water level added. The highest water level increased ivDMDig for samples with added 100 g/kg CWE to 973 g/kg DM.

## 4. Discussion

This study investigated how chestnut water extract (CWE) and quebracho (QUE) tannins, combined with increasing water levels and drying at a constant temperature, affect the in vitro degradability and digestibility of soybean meal (SBM). Overall, increasing water content consistently reduced in vitro dry matter and crude protein degradability. Tannin–protein complexes are known to form primarily through hydrophobic interactions [16] and hydrogen bonding [15], both of which are promoted in aqueous environments. Water facilitates tannin dissolution, increases molecular mobility, and supports the formation of bridging hydrogen-bond networks, enhancing the likelihood of producing complexes resistant to ruminal degradation [29,30,31,32]. Consistent with these mechanisms, ivCPDeg decreased already at the LW water level for all treatments except QUE 50, and the reduction became more pronounced at MW and HW. The samples in our study were also dried at 80 °C, which likely contributed to protein unfolding and exposure of new binding sites [33].

In accordance with our findings, Valcl and Lavrenčič [17] reported that adding water (1:2.5 *w*/*v*) and applying high temperature drying facilitated tannin–protein complexation and reduced ivCPDeg in SBM. Similar results were observed by Mezzomo et al. [18], who treated SBM with a mixture of condensed and hydrolysable tannins at 50 g/kg inclusion and added water at a 1:2 *w*/*v* ratio before drying at 55 °C, resulting in a 65% reduction in ivCPDeg. Although their drying temperature was lower than in our study, the combination of high tannin concentration and high moisture likely contributed to the decrease observed in ivCPDeg. Our results expand on these observations by demonstrating that the extent of complex formation depends strongly on the amount of water present, with lower water levels producing proportionally smaller effects. CWE consistently reduced ivCPDeg more than QUE, in line with previous work showing that hydrolysable tannins possess stronger protein-binding capacity than condensed tannins [10]. This was also confirmed by Lavrenčič and Levart [13], who observed that only 15 g CWE/kg CP were required to reduce ivCPDeg, whereas 60 g QUE/kg CP were needed to produce a comparable effect. Similar results were obtained by Li et al. [14], who found that increases in ruminal undegradable protein occurred only when fermented SBM was treated with CWE at similar water levels.

We hypothesize that these differences in tannin–protein complex formation intensity under different water levels between CWE and QUE arise from their innate molecular structure. CWE as HT contain multiple galloyl and ellagitannin units around a glucose core which together favour strong, multivalent binding to protein [31]. Their relatively lower molecular weight and more flexible structure compared with the larger, rigid proanthocyanidin polymers found in QUE improve their ability to form hydrogen bonds with soybean proteins, particularly when these proteins become partially unfolded under moist heating conditions [34,35].

In our study, QUE 50 reduced ivCPDeg only when MW and HW water was applied. This suggests that although QUE shows weaker protein binding affinity compared to CWE, binding is improved under conditions with abundant water, due to increased tannin solubility and the enhancement of both hydrophobic interactions and hydrogen bonding. Tannins primarily bind to accessible sites on the protein surface, and the high drying temperature together with abundant moisture in treated SBM likely exposed additional binding sites by disrupting and unfolding the protein structure [36,37], thus enhancing undegradable complex formation across MW and HW treatments. Taken together, these results indicate a potential to use moderate QUE concentrations with optimized water content and drying conditions to achieve desirable protection of SBM proteins potentially without impairing overall digestibility. However, this requires further in vivo confirmation.

High tannin concentrations can form complexes that are resistant to intestinal digestion [38]. In our study, CWE at ≥50 g/kg reduced ivCPDig more consistently than QUE, reflecting the stronger binding affinity of hydrolysable tannins. In CWE-treated SBM, ivCPDig increased slightly (~1%) from LW to MW/HW, where it reached a plateau below untreated control. This is consistent with reports that moist heating can partially disrupt protein structure and improve enzyme accessibility [39]. Similar small, non-significant improvements were observed when 10–50 g/kg mixed tannins were applied to moistened SBM [18]. Thus, the combined temperature and water treatment used in our study may have slightly counteracted the negative effects of CWE on post-ruminal protein digestion. In contrast, Aboagye et al. [40] reported approx. 5% lower CP digestibility with 20 g CWE/kg in an alfalfa-silage, high-forage diet, likely reflecting differences in substrate composition.

Water and tannins together also influenced bypass protein content (CP_BP) and its digestibility (dCP_BP). CP_BP increased in nearly all treatments, particularly at MW and HW, mirroring ivCPDeg responses and supporting the observations of Lavrenčič and Levart [13] and Ramaiyulis et al. [41], who reported increases in CP_BP with ≥15 g and ≥25 g tannins/kg, respectively; both studies applied water and drying. These results indicate that CP_BP is influenced not only by tannin type and concentration but also by water availability, which enhances tannin–protein complex formation and thus protects protein from ruminal degradation. dCP_BP increased when water levels were increased in treatments containing ≥50 g/kg of either tannin type. This contrasts with Cortés et al. [28], who reported a reduction in dCP_BP in SBM treated with 300–900 mg of CT/g CP. The difference could be attributed to higher tannin concentrations used, as excessive tannin inclusion forms indigestible complexes. However, our findings support the hypothesis of Valcl and Lavrenčič [17] that the presence of water during complex formation improves the proportion of bypass protein that remains digestible post-rumen. Our findings extend this hypothesis by showing that the positive effect on dCP_BP increases progressively with greater water availability, particularly at lower tannin inclusion rates.

Tannins may reduce in vitro dry matter degradability (ivDMDeg) when included at higher concentrations by impairing microbial attachment to feed particles and interfering with fibrolytic activity [7]. In our study, ivDMDeg followed ivCPDeg patterns. All tannin treatments decreased ivDMDeg except QUE 50 at LW, with stronger reductions at MW and HW. These patterns indicate that more water also enhances tannin interactions with non-protein components (e.g., fibre, starch), consistent with previous reports of 2–6% reductions with tannin extracts in SBM or mixed diets [42,43,44,45]. In our study, CWE 100 at MW and HW appeared to reach a plateau, suggesting that high tannin inclusion rates saturate most available binding sites [46].

Across treatments, effects on in vitro dry matter digestibility (ivDMDig) were limited (~2% reduction). Although statistically significant (*p* < 0.05), their biological relevance is unclear. This partially agrees with Loregian et al. [47], who reported a 7% reduction in intestinal digestibility of SBM with added 60 g tannins/kg, as well as with Valcl and Lavrenčič [17], who observed 1–2% reductions in ivDMDig with added 100 g CWE or QUE/kg under similar conditions. The ivDMDig improved for each water level beyond LW for CWE-treated samples. This suggests that CWE formed complexes with non-protein components such as starch or fibres, which may have become more digestible with increasing water availability and high temperatures [48]. Since tannins can suppress gas and SCFA production [49,50], both of which are closely linked to substrate fermentation and degradability, it is possible that our treatments also negatively affected fermentation dynamics. Further research is needed to evaluate gas production, SCFA profiles, and microbial community responses, as in vitro results may not fully reflect in vivo conditions due to microbial adaptation and post-absorptive effects.

Taken together, the degradability and digestibility results of both dry matter and crude protein indicate that the optimal water inclusion level is the medium water level (1:1.25 *w*/*v*). This water content provides substantial ruminal protein protection without imposing biologically relevant reductions in DM or CP digestibility. At MW, CWE at 50 g/kg consistently reduced ivCPDeg while maintaining ivCPDig and ivDMDig within acceptable ranges, offering the best balance between protection and nutrient utilization. QUE at 50 g/kg also became effective only at MW, providing moderate protection with minimal digestibility penalties, whereas LW was insufficient to activate QUE binding. High water content was also effective and can be used when maximum protection is desired, although the additional benefits beyond MW were marginal and did not further improve digestibility outcomes.

## 5. Conclusions

In this in vitro study we demonstrate that water availability during tannin treatment of soybean meal is a key factor of tannin–protein interactions. Increasing water content, particularly to the medium level (1:1.25 *w*/*v*), enhanced crude protein protection for both chestnut and quebracho tannins while keeping dry matter and crude protein digestibility within acceptable limits. High water content was also effective but offered only marginal additional benefits beyond the medium level. For practical formulation, SBM processed with 50 g/kg CWE at the medium water level provides the best balance between reduced ruminal CP degradability and preserved digestibility, allowing SBM to function as a higher-bypass-protein ingredient without materially compromising fermentability; 50 g/kg QUE at the medium water level is a conservative alternative when minimizing digestibility risk is the priority. At the same time, tannin inclusion reduced in vitro dry matter degradability and, to a small extent, in vitro dry matter digestibility, which may affect fermentation end products such as short-chain fatty acid and gas production. This study is limited by its fixed drying regime, reliance on in vitro data, and focus on soybean meal as the sole substrate. Future work should investigate the optimal hydration duration of soybean meal–tannin mixtures before drying, and in vivo work should validate these treatments, confirm effects on nitrogen utilization or milk protein yield on an animal level, and characterize fermentation profiles together with microbial shifts under conditions where water content, temperature, tannin type and concentration are strictly defined.

## Figures and Tables

**Table 1:** Chemical compositions (g/kg DM) of soybean meal samples after tannin and water treatments (mean ± SD).

Water Level	Tannin	Inclusion, g/kg	DM, g/kg	CP	EE	NDF	ADF	Ash
LW	NO	0	975 ± 0.3	502 ± 0.3	5 ± 0.1	125 ± 0.4	69 ± 2	69 ± 0.9
	CWE	50	973 ± 0	474 ± 5	4 ± 1	127 ± 0.7	66 ± 0.5	65 ± 0.8
		100	968 ± 5	444 ± 1	5 ± 0	154 ± 4	75 ± 1	62 ± 0
	QUE	50	961 ± 1	475 ± 1	5 ± 0.1	131 ± 5	71 ± 5	67 ± 1
		100	963 ± 3	456 ± 2	5 ± 0.1	129 ± 2	69 ± 2	60 ± 0
MW	NO	0	950 ± 2	512 ± 0.8	4 ± 0	137 ± 2	69 ± 1	65 ± 1
	CWE	50	947 ± 5	497 ± 22	5 ± 0	209 ± 3	74 ± 1	63 ± 0.6
		100	869 ± 10	446 ± 1	5 ± 0	230 ± 1	76 ± 3	63 ± 0
	QUE	50	944 ± 0.1	470 ± 0	5 ± 0	137 ± 4	72 ± 3	63 ± 0
		100	947 ± 0.4	447 ± 3	5 ± 0	135 ± 4	71 ± 0.8	61 ± 0.2
HW	NO	0	987 ± 0.3	522 ± 0	4 ± 0	146 ± 0.2	73 ± 2	71 ± 0.5
	CWE	50	988 ± 2	476 ± 3	4 ± 0.2	228 ± 0.8	74 ± 3	65 ± 0.4
		100	985 ± 0.1	438 ± 0	4 ± 0	204 ± 13	73 ± 0.1	61 ± 0
	QUE	50	976 ± 1	482 ± 1	4 ± 0.3	178 ± 19	75 ± 3	67 ± 0.1
		100	974 ± 1	461 ± 6	4 ± 0.5	142 ± 5	72 ± 0.3	64 ± 0.5

LW—water added at 1:0.625 *w*/*v*; MW—water added at 1:1.25 *w*/*v*; HW—water added at 1:2.5 *w*/*v*; NO—no tannin added; CWE—chestnut water tannin extract; QUE—quebracho tannin extract; DM—dry matter; CP—crude protein; EE—ether extract; NDF—neutral detergent fibre; ADF—acid detergent fibre.

**Table 2:** *p*-values for main and interaction effects of tannin type, concentration and water level on in vitro dry matter and crude protein degradability, digestibility, bypass protein content and bypass protein digestibility.

*p*-Value	ivCPDeg	ivCPDig	CP_BP	dCP_BP	ivDMDeg	ivDMDig
T	<0.001	<0.001	<0.001	0.052	<0.001	<0.001
C	<0.001	<0.001	<0.001	0.219	<0.001	<0.001
W	<0.001	<0.001	<0.001	<0.001	<0.001	<0.001
T × C	<0.001	<0.001	<0.001	<0.001	0.004	<0.001
T × W	<0.001	<0.001	<0.001	0.157	0.006	<0.001
C × W	0.239	0.468	0.237	0.225	0.395	0.585
T × C × W	0.440	0.006	0.389	0.007	0.599	0.571

T—tannin type, C—tannin concentration, W—water level, ivCPDeg—in vitro crude protein degradability, ivCPDig—in vitro crude protein digestibility, CP_BP—bypass crude protein content, dCP_BP—bypass crude protein digestibility, ivDMDeg—in vitro dry matter degradability, ivDMDig—in vitro dry matter digestibility.

## Data Availability

The raw data supporting the conclusions of this article will be made available by the authors on request.

## Supplementary Materials

The following supporting information can be downloaded at: https://www.mdpi.com/article/10.3390/ani16050718/s1, Table S1: In vitro crude protein degradability, digestibility, and bypass protein of SBM samples treated with different water levels; Table S2: In vitro dry matter degradability and digestibility of SBM samples treated with different water levels.

## Abbreviations

The following abbreviations are used in this manuscript:

SBM	soybean meal
HT	hydrolysable tannins
CT	condensed tannins
CWE	chestnut water extract tannins
QUE	quebracho tannins
DM	dry matter
CP	crude protein
EE	ether extract
NDF	neutral detergent fibre
ADF	acid detergent fibre
ivCPDeg	in vitro crude protein degradability
ivCPDig	in vitro crude protein digestibility
CP_BP	bypass crude protein content
dCP_BP	bypass crude protein digestibility
ivDMDeg	in vitro dry matter degradability
ivDMDig	in vitro dry matter digestibility
