# Supplementary material for: Effect of Water Level and Tannin Inclusion on In Vitro Degradability and Digestibility of Soybean Meal
Source: Animals (Basel). 2026 Feb 25;16(5):718. doi: 10.3390/ani16050718 (PMC12983951; doi:10.3390/ani16050718)
Supplement: Supplementary file 1 [file animals-16-00718-s001.zip › animals-4148542-supplementary/Supplementary Materials.pdf]

Table S1: *In vitro* crude protein degradability, digestibility, and bypass protein of SBM samples treated with different water levels

Treatments[1]			Item[2]			
Tannin	Concentration, g/kg	Water level	ivCPDeg, g/kg DM	ivCPDig, g/kg DM	CP_BP, g/kg DM	dCP_BP, g/kg DM
NO	0	LW	922[a]	997[a]	75[a]	954[a]
CWE	50		768[c]	992[c]	224[c]	963[c]
	100		640[b]	977[b]	337[b]	936[b]
QUE	50		927[a]	995[ad]	68[a]	932[a]
	100		858[ad]	995[ad]	138[ad]	965[ad]
NO	0	MW	926[a]	997[a]	71[a]	959[a]
CWE	50		690[bf]	995[acd]	305[bf]	984[bf]
	100		501[e]	984[e]	483[e]	967[e]
QUE	50		872[a]	995[ad]	123[a]	957[a]
	100		794[cd]	993[cd]	199[cd]	967[cd]
NO	0	HW	872[a]	998[a]	125[ad]	980[ad]
CWE	50		565[e]	996[ad]	431[e]	990[e]
	100		423[g]	984[e]	563[g]	972[g]
QUE	50		797[cd]	995[ad]	198[cd]	976[cd]
	100		730[cd]	994[cd]	264[cf]	976[cf]

[1]NO – no tannin added; CWE – chestnut water tannin extract; QUE – quebracho tannin extract; LW – water added at 1:0.625 w/V; MW – water added at 1:1.25 w/V; HW – water added at 1:2.5 w/V
[2]ivCPDeg – *in vitro* crude protein degradability; ivCPDig – *in vitro* crude protein digestibility; CP_BP – bypass crude protein content; dCP_BP – bypass crude protein digestibility
[a-g] - Different superscripts in the same column indicate significant differences at $p < 0.05$

Table S2: *In vitro* dry matter degradability and digestibility of SBM samples treated with different water levels

Treatments[1]			Item[2]	
Tannin	Concentration, g/kg	Water level	ivDMDeg, g/kg DM	ivDMDig, g/kg DM
NO	0	LW	941^{a}	986^{abc}
CWE	50		843^{cd}	974^{ef}
	100		770^{b}	961^{d}
QUE	50		937^{ae}	978^{abe}
	100		889^{cef}	972^{ef}
NO	0	MW	947^{a}	985^{abc}
CWE	50		801^{bd}	980^{abe}
	100		707^{g}	965^{df}
QUE	50		912^{aef}	977^{ae}
	100		868^{cf}	972^{ef}
NO	0	HW	925^{ae}	989^{c}
CWE	50		770^{b}	986^{bc}
	100		707^{g}	973^{ef}
QUE	50		862^{cf}	977^{e}
	100		843^{cd}	976^{ae}

[1]NO – no tannin added; CWE – chestnut water tannin extract; QUE – quebracho tannin extract; LW – water added at 1:0.625 w/V; MW – water added at 1:1.25 w/V; HW – water added at 1:2.5 w/V

[2]ivDMDeg – *in vitro* dry matter degradability; ivDMDig – *in vitro* dry matter digestibility

$^{a-g}$ - Different superscripts in the same column indicate significant differences at $p < 0.05$
